# Estimation of Insulin Resistance in Mexican Adults by the [^**13**^C]Glucose Breath Test Corrected for Endogenous Total CO_**2**_ Production

**DOI:** 10.1155/2012/907818

**Published:** 2012-07-17

**Authors:** Erika Ibarra-Pastrana, Maria del Carmen Candia Plata, Gerardo Alvarez, Mauro E. Valencia

**Affiliations:** ^1^Nutrition Division, Centro de Investigación en Alimentacion y Desarrollo, A.C., Mexico; ^2^Department of Medicine and Health Sciences, University of Sonora, Hermosillo, Mexico; ^3^Energy Metabolism and Body Composition Laboratory, Department of Chemical and Biological Sciences, University of Sonora, Hermosillo, Mexico

## Abstract

*Objective*. To evaluate the efficacy of the [^13^C]glucose breath test for measuring insulin resistance in Mexican adults with different glycemic states. *Research Design and Methods*. Fifty-eight adults underwent a [^13^C]glucose breath test with simultaneous measurement of total CO_2_ production by indirect calorimetry, at baseline and 90 minutes after the ingestion of 15 g of dextrose and 25 mg of [^13^C]glucose. HOMA was used as a marker of insulin resistance. *Results*. We found an inverse correlation between HOMA and the breath test **δ** ^13^CO_2_ (‰), *r* = −0.41 (*P* = 0.001). After adjusting for total CO_2_ production, correlations between HOMA and fasting glucose were less strong but remained significant. An ROC curve was constructed using **δ** ^13^CO_2_ (‰) and HOMA values; the cut-off point was 9.99‰ **δ** ^13^CO_2_, corresponding to a sensitivity of 80.0 (95% CI: 51.9, 95.7) and a specificity of 67.4 (95% CI: 51.5, 80.9). *Conclusions*. The [^13^C]glucose breath test is a simple noninvasive procedure but was not sufficiently robust for an accurate diagnosis of insulin resistance. Our findings suggest that the test might be helpful in identifying individuals who are not IR, which in turn may contribute to improved diabetes prevention.

## 1. Introduction

Obesity represents a major risk in the development of several chronic conditions, such as type 2 diabetes, hypertension, dyslipidemia, and atherosclerotic cardiovascular diseases. The precise molecular pathways underlying this close association are only partially understood [1, 2]. Type 2 diabetes is commonly preceded by a preclinical state known as pre-diabetes [3], which involves subclinical *β*-cell dysfunction known as insulin resistance (IR) [4]. Insulin resistance has been recognized as the key factor in the development of type 2 diabetes [5–7]. Moreover, data from clinical trials have shown that IR can be reduced by weight loss and changes in lifestyle behaviors and that early interventions to reduce IR can delay or prevent the onset of type 2 diabetes [8–12]. Thus, there is a need to identify individuals with IR before they develop glucose intolerance and type 2 diabetes; various methods are available, but the practical diagnosis of IR remains a challenge.

The gold standard for IR measurement is the hyperinsulinemic-euglycemic clamp [13, 14]. This method is highly sensitive; however, it requires several blood samples is invasive, expensive, time consuming, and impractical for clinical use [15]. Therefore, new simpler methods have been developed to identify stages of prediabetes, such as the homeostasis model assessment (HOMA) [16] and the quantitative insulin sensitivity check index (QUICKI) [17]. More recently, glycated hemoglobin (HbA1c) has been proposed as a diagnostic test [15]. The results of these methods have shown a strong correlation with IR as measured by the gold standard method and the oral glucose tolerance test (OGTT) [15, 17].

A novel noninvasive method has also been proposed, involving estimation of IR based on carbohydrate metabolism of ^13^C-labeled glucose [18]. In healthy individuals, insulin promotes glucose uptake by cells to be metabolized. As a product of metabolism, CO_2_ is generated and subsequently removed by the lungs through breathing [19]. Thus, when a dose of [^13^C]glucose is orally administered, the isotope tracer can be followed and quantified from a breath sample. Individuals with insulin resistance or type 2 diabetes show impaired glucose uptake and accordingly a lesser amount of expired ^13^CO_2_.

Several studies have demonstrated a good correlation between the results of the [^13^C]glucose breath test and the hyperinsulinemic-euglycemic clamp as well as with the surrogate techniques HOMA and QUICKI, in adults and children [18, 20, 21]. On the other hand, some authors have stated that a correct estimation of ^13^CO_2_ is only possible if total CO_2_ production is measured in parallel to the breath test [22], especially for IR estimation. In our review of the literature, we did not find publications where a [^13^C]glucose breath test included the measurement of total CO_2_  production.

We hypothesized that ingestion of [^13^C]glucose would produce an increase in expired ^13^CO_2_, which would provide a direct measure of insulin sensitivity, showing significant correlation with HOMA in Mexican individuals with different glycemic states. The aim of this study was to evaluate the efficacy of the [^13^C]glucose breath test to measure IR, considering total CO_2_ production in an adult Mexican population.

## 2. Research Design and Methods

This study was approved by the Ethics Committee of the Research Center for Food and Development (CIAD: Centro de Investigación en Alimentación y Desarrollo). All participants gave written informed consent before starting the protocol. Sample size was calculated based upon Pearson's correlation coefficient for association of quantitative variables, given a value of *α* = 0.05 and a power of 80% (1-**β**) [23]. The sample number was 63, but only 58 remained after excluding 2 subjects for other medical problems and 3 dropouts. This reduced the power in the order of 15%; however, the association remained highly significant. This sample was selected to potentially represent different glycemic states; 58 adult women and men (>18 years old) were allocated into three groups: healthy non-obese individuals (BMI 18.5–24.9), overweight and obese individuals (BMI ≥ 25), and newly diagnosed type 2 diabetes patients without medical treatment for diabetes [24, 25]. All subjects underwent a medical history review and physical examination by a physician to confirm eligibility. Criteria for exclusion included clinical evidence of other disorders linked to insulin resistance, such as thyroid dysfunction, acromegaly, pheochromocytoma, Cushing syndrome, and primary aldosteronism, pregnancy, and the use of drugs that could alter carbohydrate metabolism.

Subjects were asked not to perform exercise the day before the measurements and also to avoid the use of alcohol and caffeine. A venous blood sample was obtained from every subject after a 12-hour fast. Dietary intake was not determinate in participating subjects. Serum glucose and insulin were measured to calculate HOMA-IR according to Matthews et al. [16]. Insulin was measured by immunoassay (ARCHITECT Insulin, Germany). Plasma glucose, total cholesterol (TC), triglycerides (TG), and HDL and LDL cholesterol were measured immediately after the blood sample was obtained by standard methods from Randox. Estimation of HbA1c was conducted by immunoaffinity (Randox). Blood pressure (BP) was measured using an aneroid sphygmomanometer (Welch Allyn Tycos, NY, USA) according to the guidelines given by the American Heart Association [26].

Body weight (BW) was measured with subjects dressed in light clothing using an OHAUS digital electronic scale (with a capacity of 150 ± 0.05 kg). Standing height (Ht) was measured to the nearest millimeter with a Holtain stadiometer (with a capacity of 205 ± 0.5 cm), Holtain Limited, Dyfed, UK. Body mass index (BMI, kg/m^2^) was calculated based on weight and standing height. Waist circumference was measured with a fiberglass measuring tape (Lafayette Instruments Company Inc., USA) at the umbilicus level with subjects in the supine position [27].

Body composition was measured by bioimpedance analysis (BIA) (Model BIA-103, RJL System, Detroit, MI, USA). Volunteers were instructed to lie supine, with their hands at their sides and their legs separated. The skin surface was cleaned with ethanol, and the electrodes were placed on the dorsum of the right foot and hand [28, 29]. The model used to predict fat-free mass, and percentage of body fat was based on an algorithm validated for Mexican adults: FFM  (kg) = 0.7374∗(Ht^2^/*R*)+(BW) − 0.1773∗(Age) + 0.1198∗(Xc) − 2.4658, where: Ht^2^ is height in cm, *R* is resistance in ohms, BW is body weight in kg, age is in years, and Xc is reactance in ohms [28].

A breath test was performed on the same day as the other measurements. A baseline breath sample was taken, then subjects were instructed to ingest 100 mL of tap water containing of 25 mg universally labeled [^13^C]glucose (with all six carbons ^13^C) (Market Biosciences Corporation, Columbia, MD) and 15 g of USP-Dextrose, as reported in previous studies [18]. Postdose breath samples were obtained at 30, 60, and 90 minutes. To collect breath samples, the subjects were asked to expire normally through a straw into 10 mL gas Exetainer tubes (Labco Exetainer System, ^13^C and gas testing vials, Labco Limited, UK). Breath samples were analyzed by isotope ratio mass spectrometer (IRMS) (Finnigan, Breath MAT Plus, Bremen, Germany). Basal and 90-minute postdose breath samples were used to compare the absolute increase in ^13^C, expressed as *δ* in ‰  [18, 30–32].

## 3. Measurement of Total CO_2_ Production

In parallel to the breath test, we also measured total oxygen consumption (*V*O_2_), total carbon dioxide production (*V*CO_2_), metabolic rate (RMR), and respiratory quotient (RQ) using a ventilated hood indirect calorimetry unit (Deltatrac II; Sensor Medics, USA) [22, 33]. The Deltatrac gas analyzer was calibrated for each run with a span gas mixture of 95.9% O_2_ and 4.1% CO_2_. Atmospheric pressure was calibrated using an independent aneroid barometer (Cole Parmer, USA). Room temperature was kept between 24 and 26°C and relative humidity 50–60%. The Deltatrac system was regularly checked by burning known amounts of propane gas at rates similar to the subjects' energy expenditure within their body weight range. Gas burn recoveries outside the range of ±2.0% O_2_ and CO_2_ were used to adjust respiratory gas exchange of individual measurements.

Under fasting conditions, all subjects rested for at least 30 minutes at the metabolic unit, then underwent the measurement protocol. Each measurement was preceded by a 10-minute acclimatization period under the canopy; data from this period were not included in the analysis. Basal breath samples were obtained before giving the breath test solution. Deltatrac measurement was restarted, and *V*CO_2_ was permanently recorded in mL/min. Measurements were also stopped at 30, 60, and 90 minutes after the consumption of the solution to collect breath samples.

## 4. Statistical Analysis

Data are expressed as means and standard deviation. Normality was verified, and log transformation was applied when necessary and expressed as geometric means and 95% confidence intervals. A Pearson's correlation matrix was used to assess the correlation among variables; in particular, the associations between the increase of expired ^13^CO_2_ at 90 minutes after dose with HOMA and fasting insulin were analyzed before and after adjusting for the total CO_2_ production derived from indirect calorimetric measurements. The diagnostic performance of the ^13^CO_2_ method and the accuracy of the test to differentiate between diseased and normal cases was evaluated using receiver operating characteristic (ROC) curve analysis [34, 35]. The ROC curve was also used to determine the point of insulin resistance. The selected cut-off values corresponded to the highest sensitivity and specificity. Positive and negative predictor values and their 95% confidence intervals (95% CI) were obtained in the analysis, as well as the area under the curve. Using the cut-off point to define insulin resistance by ^13^CO_2_, participants were divided into insulin resistance individuals and non-insulin-resistant individuals. Clinical and metabolic variables were checked for normality and log transformed if required. Differences between these groups were analyzed using Student's *t*-test using a general statistical software (Number Cruncher Statistical System for Windows, Version 2007 Kaysville, USA). Receiver operating characteristic curves were constructed and areas under the curve (AUC) were estimated with their corresponding 95% CI by using the statistical software MedCalc 2009 (Medical Calculator, Mariakerke, Belgium). Comparison of correlation coefficients was done using Fischer's transformation to set confidence limits.

## 5. Results 

The study included 58 adult subjects (38 female and 20 male) whose clinical and metabolic characteristics are presented in Table 1.

**Figure 1:**
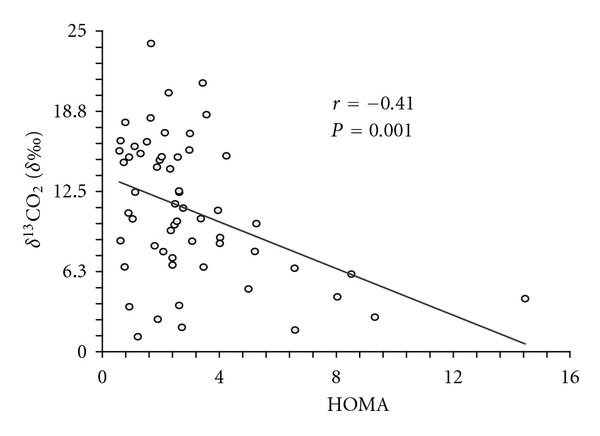
Correlation between the results of the [^13^C]glucose breath test and the Homeostasis Model Assessment (HOMA).

Figure 1 shows an inverse correlation between HOMA as a marker of insulin resistance and breath test *δ* ^13^CO_2_ (‰), with a correlation coefficient of −0.41 (*P* = 0.001) at 90 minutes after dose. Total CO_2_ production is also related to body size, and the sample included a wide range of BMI's (18.5 to 48.7); therefore, over the same 90-minute period, total CO_2_ production was also measured by a ventilated hood calorimetric method, and this data was used to correct the association between HOMA and breath test results.  Table 2 shows the correlations between *δ* ^13^CO_2_ (‰) breath test results and variables related to insulin resistance, before and after adjusting for total CO_2_ production during the test. After adjusting for total CO_2_ production, *δ* ^13^CO_2_ correlations with HOMA and fasting glucose were less strong but remained significant; however, correlations with fasting insulin, waist circumference, BMI, and percent body fat were no longer significant (*P* > 0.05).

**Figure 2:**
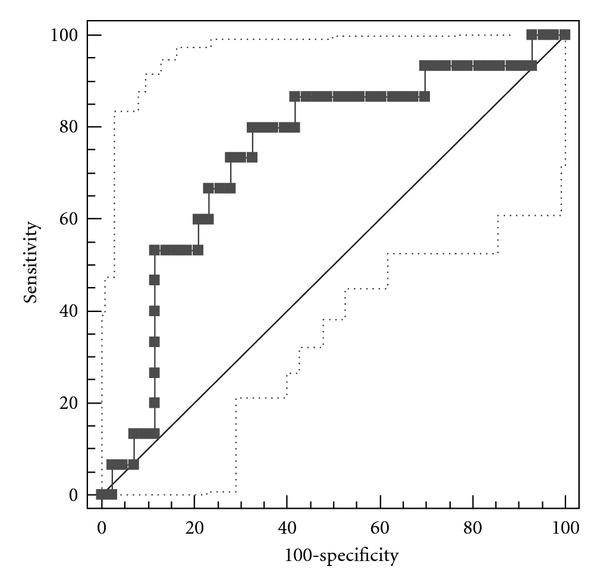
ROC (receiver operating characteristic) curve using HOMA quotient and *δ* ^13^CO_2_.

A ROC curve was constructed using *δ* ^13^CO_2_ (‰) and HOMA values, with sensitivity and specificity calculated at different levels.  Figure 2 shows the predictor ROC curve; the *y*-axis shows the sensitivity and the *x*-axis corresponds to the false positive rate of the breath test. The highest values of sensitivity and specificity were used to determine the “cut-off point” of 9.99‰  *δ* ^13^CO_2_, which corresponded to a sensitivity of 80.0 (95% CI: 51.9, 95.7) and a specificity of 67.4 (95% CI: 51.5, 80.9). Individuals below this cut-off point of 9.99‰  *δ* ^13^CO_2_ were considered insulin resistant.

Of the 58 subjects evaluated, 15 were classified as insulin resistant by the breath test *δ* ^13^CO_2_ data; 12 of these 15 were also deemed IR by HOMA results (true positives, TP), whilst 3 were not IR (false negatives, FN). Out of the 58 subjects, 43 were not classified as IR by the breath test *δ* ^13^CO_2_ data and of these results, 14 were false positive and 29 were also classified as being insulin sensitive by HOMA (true negatives, TN). 

Clinical and metabolic variables were determined to be associated with insulin resistance or insulin sensitivity based on the *δ* ^13^CO_2_ from the breath test, with a cut-off point of 9.99‰. Waist circumference and BMI were higher in individuals with IR by the breath test (*P* < 0.0001), while percent body fat was not (*P* = 0.14). HbA1c, triglycerides, and systolic and diastolic blood pressure were higher in the IR group (*P* < 0.001 and *P* < 0.02, resp.), while HDL cholesterol was lower (*P* < 0.05) and LDL differences by group did not reach significance.

## 6. Discussion 

This research focused on improving the estimation of insulin resistance using a previously proposed noninvasive method based on a [^13^C]glucose breath test [18]. In normal individuals, the presence of insulin causes glucose uptake to occur in a variety of cells, after which glucose either undergoes glycolysis and oxidation or is shunted to fat synthesis. In either case, CO_2_ is produced as a metabolic by product that enters into circulation and is eliminated by the lungs. The general assumption behind the breath test method is that ingested [^13^C]glucose will result in the expiration of detectable ^13^CO_2_. In cases of diabetes, glucose intolerance, and different stages of insulin resistance, glucose uptake would be impaired resulting in reduced rate of generation of ^13^CO_2_ [18]. 

As expected, our data showed the smallest ^13^CO_2_ values (6.03 ± 2.91  ‰) in the group of diabetic subjects, and the [^13^C]glucose breath test results showed a significant inverse association with HOMA (*r* = −0.41; *P* < 0.001). Lewanczuk et al. [18] initially compared the results of the breath test to the gold standard (the euglycemic clamp) and other surrogates methods, such as QUICKI and HOMA, in 26 subjects and found a higher correlation with the euglycemic clamp (*r* = 0.69), than with HOMA (*r* = −0.51). In 2009, Banerjee et al. [21] found a significant correlation between results of HOMA and the breath test (*r* = −0.34) in 98 subjects, which was similar to the correlation observed in the present study. Jetha et al. [20] also evaluated the [^13^C]glucose breath test in a Caucasian pediatric prepubertal sample and found a significant association between the breath test and the surrogate HOMA method (*r* = −0.51, *P* = 0.032) [20, 21]. All of these studies showed a clear relationship between the [^13^C]glucose breath test results and different IR markers, suggesting that the proposed method may be an easy and noninvasive option to diagnose IR in at-risk individuals. However, other factors must be considered. 

Dillon et al. [36] presented data from [^13^C]glucose derived ^13^CO_2_ in breath collected during a standard oral glucose tolerance test (OGTT) that was extended to a 10 h sampling period. These data showed a good separation in the appearance of ^13^CO_2_ in breath during the standard 1–3 hour oral glucose tolerance test (OGTT), averaging about 7‰  ^13^CO_2_ PDB units compared to subjects with a normal OGTT. After 4-5 hours the difference disappears. Our protocol for collecting breath samples was within this period, specifically, at 90 minutes after dose from which the AUC and cut-off points were calculated.

Rating and Langhans [37] pointed out that one important limitation of the ^13^C breath test could be endogenous CO_2_ production, which is influenced by body mass, specifically by the amount of metabolically active cells, as well by the type and quantity of macronutrients in the diet. Elwyn et al. [22] described differences between oxygen consumption and CO_2_ production in obese and normal individuals. In our study, we measured total endogenous CO_2_ production in parallel to the breath test. The total expired volume of CO_2_ at 90 minutes was used to adjust the correlation between results of the glucose breath test and HOMA. This adjustment changed the value from −0.41 to −0.26; however, these correlation coefficients were not significantly different. Our findings agree with the observations of Rating and Langhans [37] showing that the *δ* ^13^CO_2_ (‰) results depended on endogenous CO_2_ production [22, 37]. According to these authors, any breath test must take endogenous CO_2_ production into account for the calculation of actual or cumulative ^13^CO_2_ elimination or the ^13^CO_2_ peak production rate. It is possible that the correlations found in other studies might be overestimating the association between *δ* ^13^CO_2_ (‰) and HOMA or other IR reference methods. No other papers using the [^13^C]glucose breath test have also measured endogenous CO_2_. 

Sensitivity and specificity analyses were used to determine a cut-off point for the *δ* ^13^CO_2_ values (≤9.99‰  ^13^CO_2_  for IR individuals). A 2004 patent of Yatscoff et al. [38] also uses a similar cut-off point of ≤9.0‰  ^13^CO_2_; it is important to mention that the comparative methods differed between these studies. In the present study, the test accuracy was determined by the values of sensitivity (80.0) and specificity (67.4); the method mentions an observed value of sensitivity and specificity of 67, similar to our specificity value (67.4) but different from our sensitivity value of 80.

An ideal diagnostic method should have sensitivity and specificity each close to 100, and methods with values lower than 80 should not be considered [39]; however, is not common to find diagnostic methods with values above 90. In the case of ROC curves, the area under the curve (AUC) is a global measure of accuracy in a diagnostic test [39] and by itself shows the validity of the test; we found an AUC of 0.74. Morgan et al. and Swets [40, 41] stated that values between 0.5 and 0.7 indicate poor accuracy, while values between 0.7 and 0.9 suggest that the method can be used to reach certain objectives. It should be noted that for clinical applications, higher AUC values might be better indicators than high values of sensitivity and specificity together, because AUC is measure of the test accuracy; additionally AUC allows estimation of the effect size indices, which are directly related to clinical significance, particularly when predictor variables are binary. In clinical settings, screening tests with high sensitivity and relatively low specificity are more valuable when the purpose is to rapidly detect initial phases of disease. IR is a subclinical condition that precedes serious alterations of glucose metabolism; therefore, an appropriate screening strategy should prioritize early detection of IR that may delay or prevent excessive burden of type 2 diabetes. 

## 7. Conclusions

The [^13^C]glucose breath test is a simple, safe, and non-invasive method that could be used in a wide variety of individuals, including children and pregnant women. The method itself does not appear to be sufficiently robust for an accurate diagnosis of insulin resistance; however, our findings suggest that the test can be helpful to identify individuals who are not insulin resistant. This screening may contribute to allowing preventive efforts to be better addressed for the development of type 2 diabetes in clinical settings.

## Figures and Tables

**Table 1:** Clinical and metabolic characteristics of study subjects.

Variable	Diabetic	Normal weight	Overweight	Obese
Sex (F/M)	3/5	17/2	9/7	10/5
Age (years)	44.0 ± 11.4	29.5 ± 9.71	33.1 ± 12.2	41.2 ± 13.7
Weight (kg)	102 ± 34.7	59 ± 9.71	77.4 ± 10.4	92.1 ± 16.1
Height (cm)	165 ± 10.9	164 ± 6.03	168 ± 10.3	164 ± 9.94
Waist (cm)	115 ± 23.8	79.9 ± 6.68	92.4 ± 5.58	106 ± 9.94
Fat mass BIA (%)	44.2 ± 5.75	28.6 ± 11.2	35.6 ± 8.81	45.1 ± 7.68
Fasting insulin (*μ*U/mL)	22.1 ± 14.6	7.36 ± 3.85	11.0 ± 5.64	15.5 ± 8.51
Fasting glucose (mg/dL)	140 ± 56.6	87.8 ± 12.9	94.9 ± 11.1	102 ± 30.3
Triglycerides (mg/dL)	167 ± 69.4	101 ± 45.0	147 ± 86.3	192 ± 110
HDL (mg/dL)	43.5 ± 13.0	64.7 ± 16.5	55.9 ± 13.7	50.7 ± 13.1
LDL (mg/dL)	138 ± 36.8	77.3 ± 54.9	130 ± 55.8	135 ± 70.6
SBP (mmHg)	127 ± 17.9	109 ± 9.54	121 ± 19.8	139 ± 18.5
DBP (mmHg)	83.3 ± 6.18	72.2 ± 7.97	79.0 ± 10.5	87.7 ± 7.70
*δ* ^ 13^CO_2_ (‰)	7.36 ± 4.65	14.1 ± 6.21	12.0 ± 3.96	8.20 ± 3.33

Data are presented as mean ± SD. Abbreviations: F, female; M, male; BIA, electrical bioimpedance; c-HDL, high-density lipoprotein cholesterol; c-LDL, low-density lipoprotein cholesterol; SBP, systolic blood pressure; DBP, diastolic blood pressure; *δ* ^13^CO_2_ (‰), increase of ^13^CO_2_ from the basal level to that at the 90-minute time point.

**Table 2:** Correlation of *δ* ^13^CO_2_ (‰) with variables associated with insulin resistance, before and after adjustment for total CO_2_.

Variable	Before adjustment		After adjustment	
	*r*	*P*	*r*	*P*
HOMA	−0.41	0.001	−0.26	0.048
Fasting glucose (mg/dL)	−0.45	0.000	−0.37	0.004
Fasting insulin (mg/dL)	−0.27	0.035	−0.12	0.361
Waist (cm)	−0.46	0.000	−0.19	0.143
BMI (weight/height^2^)	−0.47	0.000	−0.23	0.081
Fat mass (%)	−0.29	0.024	−0.23	0.074

Abbreviations: HOMA, homeostasis model assessment; BMI, body mass index, % Fat mass, percentage of body fat mass determined by electric bioimpedance using a equation for a Mexican population.
